# Effects of a custom forelimb prosthesis on weight distribution, stability, and gait in a canine amputee: A multi-method approach

**DOI:** 10.1016/j.vas.2026.100621

**Published:** 2026-03-10

**Authors:** Aljaž Muršec, Tomaž Lampe, Dolores Sodja, Vladimira Erjavec, Miha Fošnarič

**Affiliations:** a Faculty of Health Sciences, University of Ljubljana 1000 Ljubljana, Slovenia; b Veterinary Faculty, University of Ljubljana 1000 Ljubljana, Slovenia

**Keywords:** Dog, Amputation, Prosthetics, Biomechanics, Weight distribution

## Abstract

We aimed to evaluate whether the use of a custom prosthesis improves static stability and gait symmetry in a dog with a forelimb amputation. This study involved two therapy dogs owned by the same individual, both mixed breeds with similar physical characteristics. Dog with amputation had a full forelimb amputation and had been successfully using a prosthesis since 2022. Healthy dog served as a control in three different assessment methods evaluating prosthetic effectiveness. For static measurements, a four-scale method and a force plate were used; dynamic data were collected using a pressure-sensor mat. Dog with amputation distributed 21 % of its body weight onto the prosthesis, reducing the load on the contralateral limb from 49 % to 35 %. The total forelimb load increased from 49 % to 56 % with the prosthesis. Stabilometric analysis indicated improved stability with the prosthesis: significant reduction in sway path length, while changes in directional sway components and sway area were not statistically conclusive. Sensor-mat measurements on GAITRite showed that forelimb amputation substantially reduced step length (21.1 cm) and stride length (42.4 cm) on the amputated side, and increased traversal time to 2.4 s, reflecting pronounced gait asymmetry and impaired limb function. The use of a prosthesis improved step length (35.0 and 30.1 cm) and stride length (64.1 and 63.0 cm), and decreased traversal time to 2.2 s, indicating enhanced gait efficiency, although values did not fully reach those of the healthy dog (step length 46.8/44.6 cm; stride length 93.5/87.7 cm; time 1.5 s). These results demonstrate the functional benefits of prosthetic use in canine forelimb amputation.

## Introduction

Prosthetics is a modern, clinically oriented discipline that enables the design and production of mobility devices or aids intended to assist individuals or animals with deformities, underdeveloped limbs, or traumatic and surgical amputations (Teixeira & Belinha, 2021). Nowadays, additive manufacturing technologies are becoming increasingly accessible, supporting the development of affordable and functional prostheses for animals. However, their manufacturing and application remain complex and require specialized knowledge (Kovalchuk et al., 2024). A socket prosthesis is designed so that the socket fits around the stump and anchors the prosthesis to the residual limb, while the prosthetics limb extends from the distal end of the socket to the ground, effectively replacing the missing part of the limb (Mendaza-DeCal et al., 2023). The role of prostheses is to substitute part or the entire limb after amputation, redistribute weight, protect the remaining limbs and joints from potential injuries due to the increased weight transfer onto them and potentially extend the animal’s life expectancy. However, prosthesis use also require diligent daily aftercare by the owner, including removing and cleaning the prosthesis and regularly inspect the stump for skin damage (Desrochers et al., 2014). Key aspects for a functional prosthesis for increased activity levels include body support, shock absorption, energy storage and return, and flexibility (Wendland et al., 2019). The prosthetic socket is the most critical element, as its design in the canine limb-prosthesis interface must ensure stability, load transmission, comfort, and mobility control. Most prostheses, however are passive and do not reproduce concentric muscle action (Arauz et al., 2021). The primary objectives of prostheses use include spinal alignment, reduction of weight overload on the remaining limbs, improved muscle control, and external mechanical support to promote functional independence (Silveira et al., 2022). Kinematic gait analysis techniques, which provide objective data on movement, are essential tools for understanding both normal and abnormal motion (Sandberg et al., 2020). An indirect measure of overall limb function can be obtained by measuring ground reaction forces, which indicate the distribution of weight in the measured limb during standing and walking. Designing an effective limb prosthesis for dogs requires the inclusion of biomechanical analyses to optimize and evaluate its function, with the key to replicating normal limb movement lying in the alignment of the prosthetic features with functional capabilities of the amputated dog (Arauz et al., 2021). Dogs with all four limbs distribute their body weight with 60–70 % of the total body mass borne by the forelimbs, while the hindlimbs support the remaining 30–40 % (Muršec et al., 2025). Following a forelimb amputation, dogs compensate by increasing the load on the remaining thoracic limb and, to a lesser extent on the pelvic limbs (Jarvis et al., 2013).

The aim of this study is to compare static and dynamic characteristics, such as weight distribution to the limbs, stabilometry and gait analysis, between a dog with a forelimb amputation and a healthy dog (without amputation), with a purpose to identify appropriate methods to assess the effectiveness of a forelimb prosthesis in a dog. The aim is also to compare the measurement results in the dog with an amputation, with and without the use of a prosthesis. Three different methods were used to achieve these aims.

## Materials and methods

**Fig. 1:**
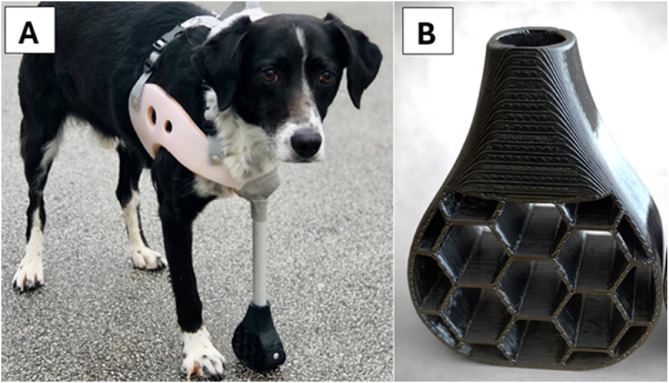
**(A)** Dog with amputation wearing a full forelimb prosthesis. **(B)** 3D-printed paw designed to absorb impact forces during movement.

The study involved two therapy dogs owned by the same person, both mixed-breed dogs with similar physical characteristics. The dog with amputation was 6 years old, weighted 16.5 kg without prosthesis, and had a height of 50 cm at the withers. This dog underwent a left forelimb amputation 5 years ago, in 2020. The reason for the amputation was extensive injury to the front left limb; no further details are known, as the dog was adopted from another country. In November 2022, it received a custom-made prosthesis from RePaw Pet (RePaw Pet s.p., Ljubljana, Slovenia), and has been using it successfully for 3 years. The prosthesis weighs 700 *g* and was made based on a plaster cast, taken by wrapping the dog's chest with plaster bandages. The prosthesis consists of a polypropylene socket, the inside of which is lined with 1 cm thick polyethylene foam. The socket is attached to the chest with four straps on the back. There are 6 holes in the socket to ensure ventilation. An aluminum rod with a diameter of 18 mm, corresponding to the length of the amputated limb, is attached to the socket. A 3D-printed paw made of flexible thermoplastic polyurethane is mounted at the distal end, designed to absorb impact forces during movement (Fig. 1A). Force absorption is possible due to the flexible material and hexagonal design, which compress under load and return to their original shape when the load is released (Fig. 1B).The second, healthy dog, serving as a control subject, was 9 years old, weighted 18.1 kg and had a height of 55 cm at the withers. Besides the forelimb amputation of one dog, both dogs were healthy and had no chronic or acute diseases. Prior to data collection, the owner signed an informed consent form.

Three methods were used to evaluate the effectiveness of the prosthesis. In the first method, four KERN scales (KERN & Sohn GmbH, Balingen, Germany) were used to assess weight distribution on each limb (each paw was positioned on a scale), including the prosthesis for dog with amputation. Each scale was 305 mm x 315 mm in size, with a maximum weight capacity of 60 kg and a resolution of ± 20 g. The scales were covered with non-slip rubber mats and placed on a flat, stable surface. Dog 1 stepped on the scales three times without the prosthesis and three times with the prosthesis, while healthy dog stepped on the scales three times. There was a two-minute break between each measurement. The distance between the scales was adjusted individually based on the dog’s standing position – measuring the distance between the front and hind limbs and the width between the limbs. The dog's posture was carefully observed to ensure correct limb placement and that the head was held straight forward and at the same height for each trial. Any head tilt or vertical displacement could distort weight distribution readings The method was carried out in the same way as in the study Muršec et al. (2025). Treats and commands from the dog's owner were used to control the posture.

**Fig. 2:**
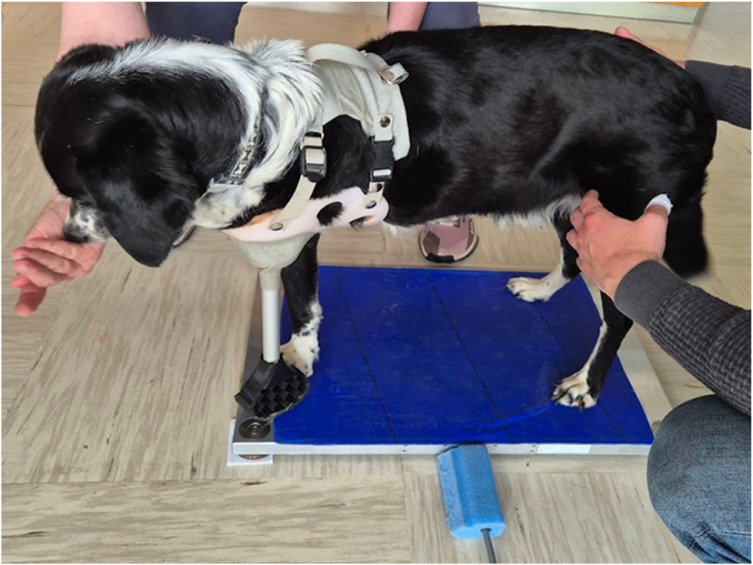
Dog with amputation standing on the pressure plate with the prosthesis in place, prior to the initiation of stabilometric measurements. The photo was taken during the first experiment, when the measurements were not yet performed. During the measurements themselves photos were not taken, as any movement of people in the room and sound can affect the results. Between individual measurements, the dogs stood independently on the plate.

In the second method, postural sway was assessed using a force platform (Kistler 9286 AA) with dimensions 60 cm x 40 cm and covered with non-slip material. Data were collected at 50 Hz sampling rate using BioWare software (Kistler Group). Similarly, as in the previously described method, dog with amputation stepped on the force plate three times with and three times without the prosthesis (Fig. 2), while healthy dog stepped on the force plate three times. Again, there was a two-minute break between trials. The dogs were positioned as similarly as possible to the first method. Each trial lasted 20 to 30 s depending on when the dog stepped out of the pressure plate on its own. From the recordings, five 10-second intervals with the static posture were selected for analysis.

**Fig. 3:**
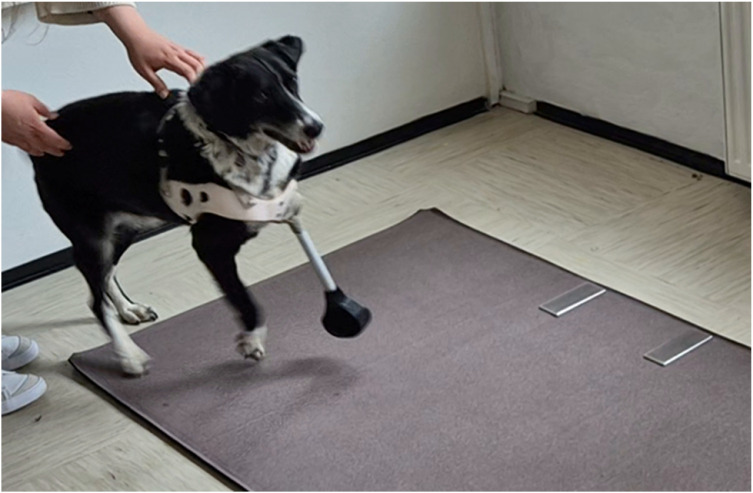
Dog with amputation equipped with the prosthesis at the starting position of the GAITRite sensor mat measurements.

The third method used a GAITRite sensor mat (CIR Systems Inc., Franklin, New Jersey, USA). The length of the mat is 525 cm. Dog with amputation walked over the mat four times, twice with and twice without the prosthesis (Fig. 3), while healthy dog walked twice. The walk began 60 cm prior to the measurement zone and ended at the end of the mat. One of the researchers stood next to the dogs at the beginning of the sensor mat, and the dog's owner stood at the end of the mat and gave the dogs the command to walk across the mat. Both dogs walked a 525 cm distance on the mat. A five-minute break was given between each measurement.

Data from the first method (four scales) and the third method (sensor mat) were processed using Microsoft Excel 2016 software (Microsoft, Redmond, WA, USA). Data obtained from the second method (pressure plate) were analyzed using StabData software (*Https://Www.Zf.Uni-Lj.Si/Images/Ri/Publikacije/Starostniki2006/10_SevsekRugelj.Pdf*, n.d.) to obtain the following stabilometric outcome measures for 10-second (*t*= 10 s) measure intervals:

• total path length of the center of pressure (CoP), calculated by connecting all measured CoP points (by sampling rate of 200 Hz we obtained 2000 CoP points for each measure interval);
• path lengths of CoP in medio-lateral (M-L) and in cranio-caudal (C—C) directions (i.e. projections of the total path length to M-L or C—C axis, respectively);
• estimations of the total sway area of CoP using Fourier Analysis (FAO) and Principle Component Analysis (PCA) methods (*Https://Www.Sevsek.Eu/Bibliografija/Clanki/WSEAS_Transactions_2007.Pdf*, n.d.):

Obtained stabilometric outcome measures were statistically evaluated using Python in JupyterLab IDE SciPy (scipy.stats) and NumPy libraries. For the amputated dog, we assessed the within-subject effect of the prosthesis by comparing outcome measures without prosthesis vs. with it, across the five matched measure intervals (10 s each) using paired tests. For each outcome measure, we first tested normality of the paired differences using the Shapiro–Wilk test (*α* = 0.05). If differences were approximately normal, we used a paired *t*-test and reported the mean difference (with − without prosthesis), 95 % confidence interval (CI), *p*-value, and Cohen’s *d_z_* as an effect size. If normality was not satisfied, we used the Wilcoxon signed-rank test (two-sided) and reported the *p*-value and rank-biserial correlation as an effect size. All hypothesis tests were two-sided and evaluated at a significance threshold of *α* = 0.05 (*p*< 0.05). Due to single-subject groups, comparisons involving the healthy dog were treated as descriptive (mean ± SD).

## Results

**Fig. 4:**
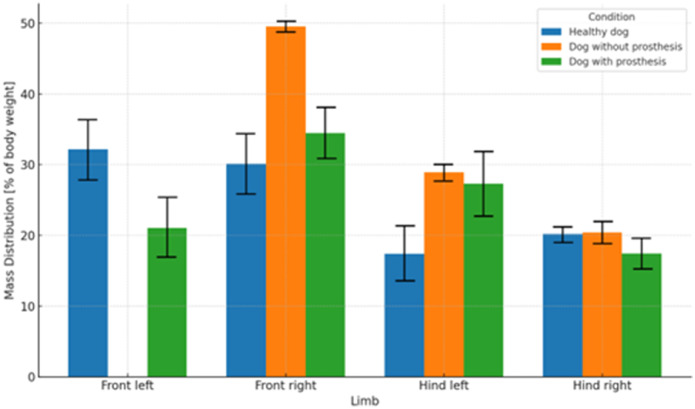
Comparison of limb weight distribution for a healthy dog ​ and a dog with amputation, evaluated both with and without the prosthesis.

The weight distribution on each limb, including the prosthesis of dog with amputation, was assessed using four scales. The average percentage of weight born by each limb and the prosthesis was calculated from the individual measurements (Fig. 4). The healthy dog distributed its weight almost evenly between the forelimbs (left 32 %, right 30 %). Dog with amputation and without the prosthesis, placed 49 % of its body weight on the healthy forelimb, while with the prosthesis, it placed 35 % on the healthy forelimb and 21 % on the prosthesis.

For dog with amputation, the use of the prosthesis significantly altered the weight distribution on the hind limbs. Without the prosthesis, it distributed 49 % of its weight to the forelimb and 51 % to the hind limbs. With the prosthesis, it distributed 56 % to the forelimb and prosthesis, and 44 % to the hind limbs. For comparison, healthy dog distributed 62 % of its body weight to the forelimbs and 38 % to the hind limbs.

**Fig. 5:**
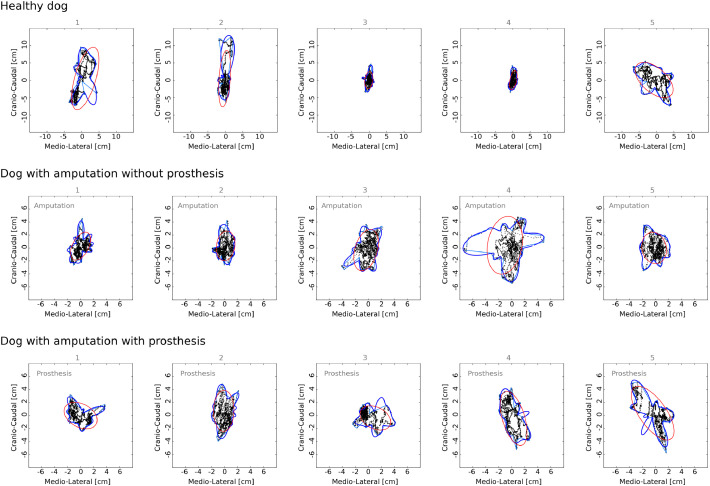
Stabilograms for healthy dog and for dog with amputation without and with the prosthesis, across five (*N*= 5; indicated above each stabilogram in gray) measurement intervals of 10 s each. Black dots are positions of the centre of pressure (CoP). With a sampling rate of 200 Hz, there are 2000 measured CoP positions on each stabilogram. The borders of the estimated CoP sway area are shown for FAO method (blue; for comparison, light blue lines indicate an approx. convex hull of CoP points) and PCA method (red). The horizontal and vertical axis of stabilograms represent medio-lateral and cranio-caudal directions, respectively. The position of the amputation/prosthesis of the left forelimb is indicated on the stabilograms (gray).

**Fig. 6:**
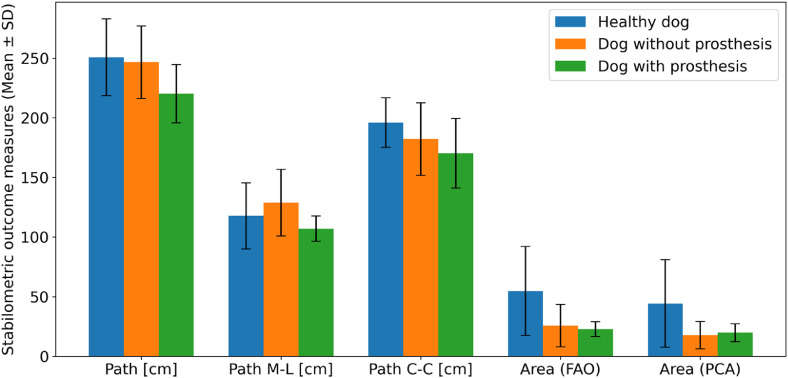
Comparison of stabilometric outcome measures for healthy dog (blue bars), and dog with amputation without prosthesis (orange bars) and with prosthesis (green bars). Bars show mean values across five 10-second measurements for stabilometric outcome measures: path of the centre of pressure (CoP), path of CoP in medio-lateral direction (M-L), path of CoP in cranio-caudal direction (C—C), and CoP sway area estimated with FAO and PCA methods. Error bars indicate standard deviations.

For illustration of results of stabilometric measurements on the pressure plate, Fig. 5 shows stabilograms with trajectories of the center of pressure (CoP) for each measure interval (5 per dog and condition). Fig. 6 shows the mean values and standard deviations of the outcome measures describing the sway of CoP over five measuring intervals for each dog and condition.

Data from the healthy dog were summarized descriptively (Fig. 6), without formal statistical testing due to the single-subject design. For a dog with amputation, paired analysis across five matched measuring intervals revealed that wearing a prosthesis significantly reduced total CoP path length. For total path of CoP, the paired *t*-test indicated a mean difference of −26 cm (with − without prosthesis), 95 % CI [−47, −5.6], *t*(4) = −3.5, *p*= 0.025, with a large effect size (Cohen’s *d_z_* = −1.6). This corresponds to an approximate 11 % decrease in CoP path length when the prosthesis was worn.

For path of CoP in medio-lateral direction, the reduction did not reach statistical significance (*t*(4)=−1.6, *p*= 0.18), although the mean difference was −22 cm (95 % CI [−59, 15]) with a moderate effect size (*d_z_* = −0.73).

For path of CoP in cranio-caudal direction, the Wilcoxon signed-rank test showed no significant change (*W*= 1.0, *p*= 0.13), with a mean difference of −12 cm and a rank-biserial correlation of −0.60 (moderate effect).

For CoP sway area estimated with FAO method, Wilcoxon test yielded *W*= 5.0, *p*= 0.63 (mean difference = −2.9; rank-biserial = 0.60). For Area (PCA), the paired *t*-test indicated *t*(4) = 0.31, *p*= 0.77, with a trivial effect size (*d_z_* = 0.14) and a mean difference of +2.0 (95 % CI [−16, 20]).

Overall, the prosthesis was associated with a robust decrease in total CoP path length, while changes in directional components and CoP area were not statistically conclusive. Effect sizes and confidence intervals are reported to aid interpretation beyond *p*-values.

The third method for evaluating prosthesis effectiveness used the GAITRite sensor mat. Both dogs walked a 525 cm distance on the mat. The average results of time, step length and stride length measurements are shown in Table 1.

**Table 1:** Measurements of time, stride length and stride length on the GAITRite sensor mat in a healthy dog and a dog with an amputation with and without a prosthesis.

Sensor mat results	Healthy dog (without amputation)		Dog with amputation without a prosthesis		Dog with amputation with a prosthesis	
Time (s)	1.5		2.4		2.2	
Limb side	Right	Left	Right	Left	Right	Left
Step length (cm)	46,8	44,6	21,1	/	35,0	30,1
Stride length (cm)	93,5	87,7	42,4	/	64,1	63,0

Based on the sensor-mat measurements, the dog without a prosthesis shows a reduction in both step length and stride length, together with an increased time required to traverse the mat. Compared with the healthy dog, the dog with amputation without a prosthesis demonstrates a substantially shorter step length (21.1 cm) and stride length (42.4 cm), indicating pronounced gait asymmetry and compromised limb function. With the prosthesis, the dog exhibits notable improvement: both step length (35.0 and 30.1 cm) and stride length (64.1 and 63.0 cm) increase toward values closer to the healthy dog, and traversal time decreases from 2.4 s to 2.2 s, suggesting enhanced gait efficiency. Although these values do not fully reach the performance of the healthy dog (step length 46.8/44.6 cm; stride length 93.5/87.7 cm; time 1.5 s), the prosthesis clearly reduces the magnitude of gait deficits.

## Discussion

This study investigated the differences in static and dynamic weight distribution between a dog with a forelimb amputation fitted with a custom-made prosthesis and a healthy control dog.

The results of weight distribution using the four-scales method showed that the dog with amputation and without prosthesis carried 49 % of its body weight on the intact forelimb and 51 % on the hind limbs, with the ipsilateral hind limb bearing more weight (29 %). The healthy control dog carried 62 % on the forelimbs and 38 % on the hind limbs. Several studies report that the normal weight distribution in healthy dogs is approximately 60 % on the forelimbs and 40 % on the hind limbs (Carr et al., 2018, Goldner et al., 2015, Rodriguez et al., 2024; Wendland et al., 2023).

Similar results were obtained in the study by Cole and Millis (2017), where ten dogs with forelimb amputations were examined under static conditions. The results showed that approximately 47.5 % of the body weight was transferred to the intact forelimb. Similar results were reported by Filho et al. (2024), where dogs with high-level forelimb amputations under static conditions carried 50.7 % of their weight on the intact forelimb, with an increased load on the ipsilateral hind limb (27.9 %).

Also the study by Ben-Amotz et al. (2020) found that dogs compensate for forelimb amputation by shifting their weight to the ipsilateral hind limb. Jarvis et al. (2013) reported a 14 % increase in weight bearing on the intact forelimb and a 17 % increase on the hind limb compared to controls. On average, dog with amputation carried 56 % of their body weight on the hind limbs, while healthy dogs carried 40 %. Kirpensteijn et al. (2000) found that dogs with forelimb amputations carried 46.9 % of their body weight on the intact forelimb and 53.1 % on the hind limbs, while control dogs distributed 59.8 % on the forelimbs and 40.2 % on the hind limbs.

The results of the four-scales method showed that dog with amputation transferred 21 % of its body weight to the prosthesis, reducing the load on the intact forelimb and both hind limbs. Use of the prosthesis reduced the hind limb load from 51 % to 44 % and the load on the intact forelimb from 49 % to 35 %. With the use of a prosthesis, a more physiological weight distribution (60–40 %) was achieved, as the dog supported 56 % of its body weight on the forelimbs and 44 % on the hind limbs. Similarly, in the study Wendland et al. (2023) also investigated weight distribution in dogs with partial forelimb amputations that were fitted with the prosthetic devices. Their study included ten dogs, and the prostheses supported an average of 26 % of body weight.

Based on the obtained results (Fig. 4) and previous studies, it can be concluded that forelimb prostheses contribute to an improved distribution of body weight; however, this redistribution is not complete, as prostheses bear a lower load compared to a healthy forelimb. Furthermore, based on the results and comparison with previous studies, it was found that dogs with a forelimb amputation without the use of a prosthesis transfer a greater proportion of body weight to the hind limbs, particularly to the ipsilateral hind limb. Even with the use of a prosthesis, a greater load is still borne by the ipsilateral hind limb. It can therefore be concluded that, due to the amputation and medio-lateral stability requirements, the healthy (hind) limb on the affected side of the body bears an increased load.

Comparing stabilometric measurements across dogs is challenging due to variability in their ability to stand still; however, within-subject comparisons were feasible. In our study, we observed a significant reduction in sway path length when the prosthesis was used, suggesting improved stability and supporting its role in balance control. Changes in directional sway components and sway area were not statistically conclusive, and confidence intervals and effect sizes were reported alongside *p*-values to address limitations of small-sample inference. These findings highlight the complexity of interpreting sway metrics, as smaller excursions may reflect stability, reduced mobility, or simply the ability to stand still. Future studies with larger samples and complementary functional measures are needed. Normalization to body height is generally recommended, but the 10 % height difference between our two dogs likely had minimal impact.

A similar method was used in the study Rodriguez et al. (2024) analysed static and dynamic parameters in five French Bulldogs with forelimb amputations and five healthy dogs of the same breed. Using a force plate, they found that forelimb amputation did not affect the overall stability but increased the risk of injury to the remaining limbs. In contrast, Izawa et al. (2021) reported different results. In their study of six Beagle dogs, force plate measurements showed that when standing on all four limbs, the body's centre of gravity was positioned cranially to the body’s midpoint. However, when one forelimb was lifted, the centre of gravity shifted caudally and contralaterally.

The results on the GaitRite sensor mat illustrate functional benefits of prosthetic use in a dog with limb amputation. As expected, the absence of a prosthesis leads to pronounced gait asymmetry, reduced step and stride lengths, and prolonged traversal time, reflecting compensatory unloading and altered locomotor mechanics. With the prosthesis, these deviations are partially corrected, demonstrating improved limb engagement and more efficient gait. Although the prosthetic-assisted gait does not fully match the performance of the healthy dog, the measurable improvements support the prosthesis as an effective aid for restoring more symmetrical locomotion. Further studies with larger sample sizes and repeated trials would be needed to confirm these findings statistically. The same findings were reached in the study by Fuchs et al. (2014). The authors investigated gait dynamics in dogs before and after simulated hind limb loss on a treadmill. The results showed that when walking with three limbs, the step and stride length decreases, also walking speed. In summary, the use of a custom forelimb prosthesis reduced the load on the intact limbs, improved weight distribution, and showed signs of improved static stability and gait efficiency. While statistical significance was not achieved in some parameters, the observed trends support the potential functional benefit of prosthetic use in canine amputees.

Forelimb amputations and associated musculoskeletal injuries can lead to significant biomechanical imbalances. Without intervention, dogs may develop compensatory deformities in the remaining limbs, including altered joint angles, muscle contractures, and asymmetrical limb loading. Over time, these imbalances can contribute to secondary conditions such as C-shaped scoliosis, uneven spinal curvature, and increased stress on contralateral limbs, which may predispose older or heavier dogs to osteoarthritis, joint degeneration, or chronic pain Arauz et al. (2021).

Based on the results obtained from the three methods and previous studies, we believe that the use of prostheses in dogs significantly improves biomechanics, thereby reducing the risk of secondary injuries and deformities in the remaining limbs caused by overload and abnormal weight distribution during both static and dynamic activities. Prosthetic intervention supports proper limb alignment, restores a more physiological gait, and redistributes body weight more evenly. By mitigating excessive stress on the remaining limbs and spine, prostheses help prevent further deformities, promote long-term musculoskeletal health, improve mobility, and enhance overall quality of life.

Small sample size (one amputee and one healthy dog) limit the generalizability of our findings. Further studies with larger cohorts are necessary to confirm these trends. We also believe that different levels of forelimb amputation and variations in prosthesis design could produce different outcomes. We hypothesize that dogs with more distal amputations, in which more joints are preserved, would achieve better results. Another limitation of this study is the limited number of previous studies available for comparison with our findings.

## Conclusions

Animal prosthetics is not a widely described field, as it is still developing globally, as shown by the limited number of studies. Each animal case differs due to the many breeds and mixed varieties, so each animal must be treated individually. This study provides preliminary evidence supporting the functional benefits of forelimb prosthesis use in dogs following complete limb amputation. The prosthesis enabled the affected dog to redistribute part of its body weight onto the prosthesis (21 %), thereby reducing excessive load on the contralateral forelimb from 49 % to 35 %. This suggests a potential for improved musculoskeletal balance and a decreased risk of secondary overuse injuries. Additionally, gait analysis revealed that the use of the prosthesis resulted in a more efficient walking pattern, characterized by increased walking speed, step and stride length. While stabilometric data showed a trend toward improved stability with the prosthesis, statistical significance was not achieved for all outcome measures, likely limited by the small sample size. Further studies involving larger cohorts and long-term follow-up are needed to confirm these findings and to refine prosthetic design and rehabilitation strategies in veterinary orthotics and prosthetics. Nevertheless, the results of this case-based investigation highlight the promising role of prosthetic devices in enhancing mobility, load distribution, and functional recovery in dogs.

## Funding statement

This work was supported by the Faculty of Health Sciences and the Veterinary Faculty of the University of Ljubljana.

## Data statement

Research data supporting the findings of this study are available upon request.

## CRediT authorship contribution statement

**Aljaž Muršec:** Writing – original draft, Visualization, Software, Resources, Methodology, Investigation, Data curation, Conceptualization. **Tomaž Lampe:** Writing – review & editing, Supervision, Project administration. **Dolores Sodja:** Writing – original draft, Investigation. **Vladimira Erjavec:** Writing – review & editing, Visualization, Validation, Supervision, Funding acquisition, Formal analysis. **Miha Fošnarič:** Writing – review & editing, Software, Methodology, Investigation, Funding acquisition, Data curation, Conceptualization.

## Declaration of competing interest

Aljaž Muršec: I have The authors declare that they have no known competing financial interests or personal relationships that could have appeared to influence the work reported in this paper.
